# Molecular basis of *DYT1* and *DYT6* primary dystonia in Indian patients

**DOI:** 10.1186/1755-8166-7-S1-P121

**Published:** 2014-01-21

**Authors:** Subhajit Giri, Arindam Biswas, Shyamal Kumar Das, Kunal Ray, Jharna Ray

**Affiliations:** 1 S. N. Pradhan Centre for Neurosciences, University of Calcutta, Kolkata, India; 2 Bangur Institute of Neurosciences & Psychiatry, Kolkata, India; 3 CSIR-Indian Institute of Chemical Biology, Kolkata, India

## Background

Dystonia is the third most common movement disorder. It is manifested by involuntary and sustained muscle contractions with frequent twists and repetitive movement, abnormal posture and functional impairment. Genetic factors play significant role for causing dystonia. Till date twenty five loci (*DYT1-DYT25*) and sixteen genes have been reported for dystonia. The present study reports the screening of TOR1A (*DYT 1*) and THAP1 (*DYT 6*) among primary torsion dystonia patients of India.

## Materials and methods

First, the most common ΔGAG mutation (c. 904-906/907-909 ΔGAG; p. Glu302/303del) and the rs1801968 (p. Asp216His) in TOR1A gene were screened following the published method (Naiya et al., 2006). *THAP1* was screened in 214 patients to identify mutations (mean age of onset, 30.8 ± 16.42 years) and 254 controls (mean age, 41.9 ±11.59 years). All three exons and their flanking sequences including exon-intron boundaries were screened by PCR , sequencing and/or RFLP analysis.

## Results

A total of 321 patients were screened and ΔGAG mutation was identified in two brothers in a family suffering from primary generalized dystonia. The analysis of rs1801968 (Asp216His) demonstrated that the minor allele (216His) is significantly over represented in patients (p = 0.027; OR = 1.915; 95% CI = 1.058-3.451), suggesting a risk for the disease. On screening *THAP1* in 214 patients, a total of five nucleotide variants were identified including a reported missense mutation (c.427 A>G; p.Met143Val), a novel heterozygous deletion mutation (c. 208-209 del AA; p. K70VfsX15) in two juvenile onset primary dystonia patients and a rare variant in 3ʹ UTR region (c. 1030 T>C) in a patient having Blepharospasm. In addition two SNPs, (rs71521601 and rs111989331), were also found both in patients and controls. The association study using rs111989331 (IVS2-87 A>G) demonstrate that the major allele (A) can play as a risk factor (p = 0.001; OR = 1.977; 95% CI = 1.302-3.008) for primary dystonia.

## Conclusion

Our preliminary results suggest that the *TOR1A* and *THAP1* genes play significant role in the pathogenesis of Indian dystonia patients. This is the first report on mutation detection in TOR1A and THAP1 among Indian dystonia patients.

